# Conservation of reef manta rays (*Manta alfredi*) in a UNESCO World Heritage Site: Large-scale island development or sustainable tourism?

**DOI:** 10.1371/journal.pone.0185419

**Published:** 2017-10-25

**Authors:** Steven Thomas Kessel, Nasreldin Alhasan Elamin, David James Yurkowski, Tarik Chekchak, Ryan Patrick Walter, Rebecca Klaus, Graham Hill, Nigel Edward Hussey

**Affiliations:** 1 Daniel P. Haerther Center for Conservation and Research, John G. Shedd Aquarium, Chicago IL, United States of America; 2 Equipe Cousteau, Paris, France; 3 Wildlife Conservation General Administration, Port Sudan, Sudan; 4 Great Lakes Institute for Environmental Research, University of Windsor, Windsor, ON, Canada; 5 Senckenberg Research Institute and Museum of Nature Frankfurt Marine Zoology / Ichthyology Senckenberganlage 25, Frankfurt A.M., Germany; 6 The Deep Aquarium, Hull, United Kingdom; 7 Biological Sciences, University of Windsor, Windsor, ON, Canada; Department of Agriculture and Water Resources, AUSTRALIA

## Abstract

A large reef manta ray (*Manta alfredi*) aggregation has been observed off the north Sudanese Red Sea coast since the 1950s. Sightings have been predominantly within the boundaries of a marine protected area (MPA), which was designated a UNESCO World Heritage Site in July 2016. Contrasting economic development trajectories have been proposed for the area (small-scale ecotourism and large-scale island development). To examine space-use, Wildlife Computers^®^ SPOT 5 tags were secured to three manta rays. A two-state switching Bayesian state space model (BSSM), that allowed movement parameters to switch between resident and travelling, was fit to the recorded locations, and 50% and 95% kernel utilization distributions (KUD) home ranges calculated. A total of 682 BSSM locations were recorded between 30 October 2012 and 6 November 2013. Of these, 98.5% fell within the MPA boundaries; 99.5% for manta 1, 91.5% for manta 2, and 100% for manta 3. The BSSM identified that all three mantas were resident during 99% of transmissions, with 50% and 95% KUD home ranges falling mainly within the MPA boundaries. For all three mantas combined (88.4%), and all individuals (manta 1–92.4%, manta 2–64.9%, manta 3–91.9%), the majority of locations occurred within 15 km of the proposed large-scale island development. Results indicated that the MPA boundaries are spatially appropriate for manta rays in the region, however, a close association to the proposed large-scale development highlights the potential threat of disruption. Conversely, the focused nature of spatial use highlights the potential for reliable ecotourism opportunities.

## Introduction

Anthropogenic disturbances, in various forms, are the largest driver of conservation concerns for global species [1]. On the local scale, disturbances confined to discrete habitats have considerable impacts on sessile or low-vagility species, but are often assumed to have reduced influence on highly mobile animals [2]. This assumption stems from the perception that mobile species can select alternative areas, but does not hold true when a group of animals have a disproportionate reliance on a specific area that is impacted [3]. Determining the relative importance of habitats for mobile species is a prerequisite to assessing the effectiveness of current or proposed conservation measures, such as marine protected areas (MPAs), in ensuring population persistence [3]. From a management perspective, the identification of key habitats of mobile species also provides a more feasible management option than attempting to protect their entire home range [4]. The success of management options such as MPAs, however, is dependent on the spatial appropriateness of the designated boundaries of the protected area relative to the life history of the focus species, in addition to effective enforcement measures.

The reef manta ray (*Manta alfredi*) is a large, highly *k*-selected species for which there is growing concern over the status of global populations [5], with several long-term monitoring studies noting declines in animal sightings [6–8]. The primary cause for concern has been fisheries impacts, from both incidental and directed captures, with manta rays targeted mainly for their branchial filter plates for use in traditional medicines [8–10]. As a result, reef manta rays are currently listed as ‘Vulnerable’ under the IUCN Red List, and in 2013 were listed on Appendix II of the Convention on International Trade in Endangered Species (CITES [11]). Unlike the pelagic manta ray (*Manta birostris*), reef manta rays display a high level of association with shallow coastal waters, particularly lagoon areas where notable aggregations of this species have been documented globally across tropical and subtropical regions [3, 4, 12].

**Fig 1:**
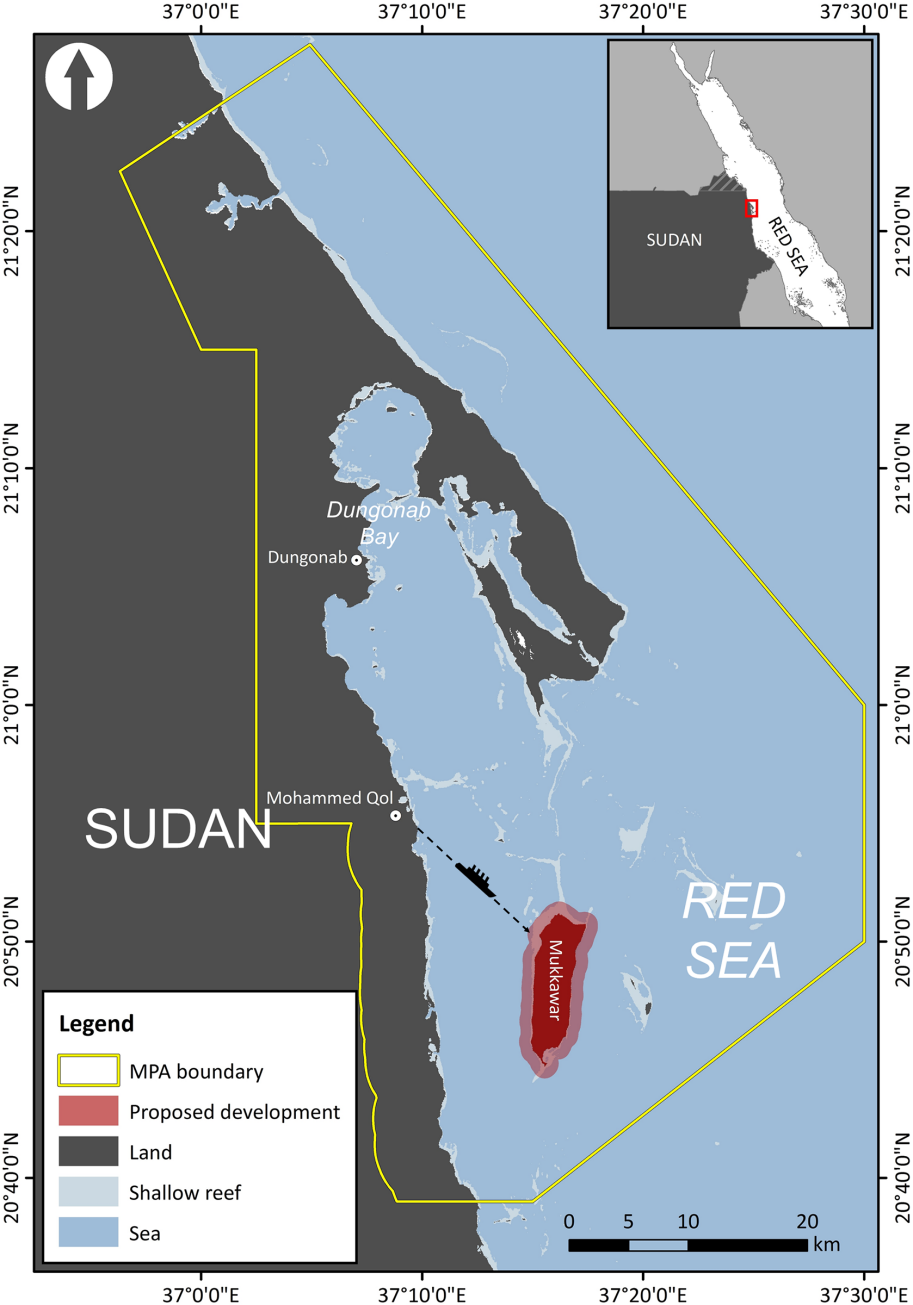
Dungonab Bay and Mukkawar Island National Park (DMNP), located on the western shore of the Red Sea. Dotted circles show locations of Beja communities located within the MPA. Dashed line and ship icon indicate the most probable shipping traffic route from the main land, based on ease of coastal access. Inset shows location of Dungonab Bay on Sudan’s Red Sea Coast.

The north Sudanese coast of the Red Sea is one such habitat, where a large reef manta ray aggregation commonly occurs [13, 14], yet almost no focused research has been conducted to date (but see [15, 16]). The majority of reef manta ray observations occur within the boundaries of Dungonab Bay and Mukkawar Island National Park (DMNP; Fig 1), an area designated a MPA in 2004 and declared a UNESCO World Heritage Site in 2016 [17]. Hass [18] reported sighting 40 individuals to the north of Mukkawar Island during his expedition to the Red Sea in the 1950s. Between 19–30 June 2006, groups of up to eight individuals were observed feeding around reefs north of Mukkawar Island [19]. In November 2007, surveys recorded groups of mantas in the same vicinity, with more than 40 individual recorded in groups of between five to six animals, including males, females, and juveniles [20]. These manta rays are commonly observed forming feeding trains in plankton-rich waters [16, 21] and are a focus of seasonal live-a-board dive tourism. Dungonab Bay may serve as a nursery ground for manta rays, with adult, juvenile and neonate animals observed [20], and it is the site of the first, and to date only, documented *Manta alfredi* × *Manta birostris* hybrid [15]. As such, Dungonab Bay could be considered priority habitat for reef manta rays in the Red Sea region and, despite difficulties associated with conservation efforts in politically unstable regions [22], further study is required for regional and global management of this large manta aggregation site.

A proposed large-scale island development, ‘The Heart of the World’, to be built within the boundaries of the DMNP on Mukkawar Island (Fig 1), has raised concern over the critical need for information to assess potential development impacts on this localised manta ray aggregation. The proposed plan identifies intense development of the terrestrial environment, including an international airport and the world’s tallest skyscraper [23]. Furthermore, planned mass seabed evacuation and land reclamation, on a scale similar to Nakheel marine development projects in Dubai that saw >200 million m^3^ of marine sediment dredged [24], would be undertaken. This proposed large-scale development, commanding a potential US$11 billion investment, has been discussed in the newspapers and at the government level in Sudan. Important government stakeholders, such as the Ministry of Environment, Natural Resources and Physical Development and Wildlife Conservation General Administration (WCGA), are more in favour of lighter sustainable tourism development. Coastal development can impact marine populations through loss of critical habitat [25], acoustic disturbance [26], increased pollution [24], increased anthropogenic activities related to tourism [24], physical disturbance [27] and reduced survival rates of nursery populations [28]. Of particular concern for reef manta rays is greatly increased turbidity as a result of dredging, which can decrease primary production potential and reduce the planktonic food resources in the area [29–31]. Issues associated with development can often be exacerbated in developing countries due to corruption, a lack of development regulations and official precautionary procedures such as environmental impact assessments (EIAs), and in turn associated mitigation measures [32, 33].

To assess the effectiveness of spatial management measures, such as MPAs, it is necessary to investigate the relative extent of species movements and define their core use areas. One tool that can be employed for this purpose on larger species is satellite telemetry (e.g. [34]). Positional satellite tags can provide near real-time location estimates for individual animals that commonly surface, allowing quantification of horizontal space use [35]. This telemetered monitoring technique is a particularly desirable method in logistically complex regions, such as Sudan, as once the animal is tagged all data is relayed autonomously through satellite, minimizing required field time and maintenance of *in situ* equipment.

Given the concern over global reef manta ray populations, and the potential importance of this regionally unique aggregation site, this study aimed to provide baseline information on the spatial habitat use of manta rays in Dungonab Bay, relative to the MPA boundaries and the proposed large-scale development. Specifically, through the use of near real-time satellite telemetry, this study aimed to i) assess the spatial effectiveness of the MPA for protecting reef manta ray habitat; and ii) investigate the level of association between animal movements within the MPA and the proposed site of the large-scale island development site.

## Methods

### Ethics statement

This study was conducted under the approval of the University of Windsor's Institutional Animal Care and Use Committee (IACUC; AUPP # 13–08), and under field permissions provided by the Wildlife Conservation General Administration of Sudan.

### Study site

Dugonab Bay (20° 52’ N, 37° 14’ E) is located on the north eastern coast of Sudan, on the western shore of the Red Sea (Fig 1). The DMNP encompasses a 2,957 km^2^ area that contains several small islands and one large island, Mukkawar (30 km^2^, Fig 1). Depths within the MPA extend down to ~50 m in some locations, with shallower areas typically ~3 to 5 m throughout. The shallow areas contain rich strip and patch reefs that are high in biodiversity and biomass, whereas the adjacent terrestrial coastal area and islands are desert. Mean chlorophyll-a and turbidity remains very consistent throughout the year in the Dungonab Bay region (supplementary information S1, S2 and S3 Figs). Rainfall on the Red Sea coast of Sudan is variable but low given the arid nature of the environment. Total annual rainfall on the coastal plain is around 75 mm with an average monthly rainfall ranging from <1mm to a maximum of 30–35 mm per month during the winter months (October to January) with peak rainfall in November. This area was designated as a MPA in response to the richness and diversity of species contained within, including coral reefs, dugongs, sea turtles (including nesting sites), birds and elasmobranchs, specifically manta rays [13, 14]. Recently, the outstanding ecological importance of this site has led to its inscription as a World Heritage Site [17].

### Capture and tagging

Reef manta rays were targeted inside the MPA boundaries using a novel capture method. For the first time, mantas were physically captured for processing, rather than tagged while free swimming using a tagging pole or spear gun. Individuals were captured by a hook and line rig consisting of a 2 m long aluminium pole, tipped with a 20/0 break-away circle hook attached to a 30 m length of 1.5 cm diameter twisted nylon rope, with two 20 litre plastic cooking oil containers attached as floats. Individuals swimming at the surface were approached slowly from the rear in a 5 m fiberglass vessel. When in range, the aluminium pole was manoeuvred over the head to position the hook in the centre of the palatoquadrate. At the moment of hooking, the individual would dive and increase swimming speed, and as the rope spooled out of the boat, the floats were thrown overboard. Hooked individuals were left to tow the floats for ~15 mins, until sufficiently fatigued to facilitate greater ease of manipulation. Following this period, the individual was manoeuvred to the side of the boat by hand, and a rope was placed over the tail and dorsal fin to secure the animal along-side the vessel for tagging. Wildlife Computers^®^ SPOT 5 tags were secured to the dorsal fin using nylon bolts at four points of attachment (see supplementary information S4 Fig for attachment image). The tags were programmed to transmit a maximum of 250 times a day. The sex of the individual was recorded, the wingspan measured to the nearest 0.5 cm, and a small piece of tissue was removed from the posterior edge of the dorsal fin for genetic analysis, which confirmed all tagged individuals as *Manta alfredi*. Post tagging, the hook was removed and the animal released and monitored by a snorkeler until it was no longer in sight.

### Data analysis

A two-state switching Bayesian state space model (BSSM) was fitted to the SPOT tag data that allows movement parameters to switch between two behavioural states, resident and traveling, in discrete time and within a hierarchical framework to improve parameter and location estimation [36]. The BSSM was implemented using the package bsam v0.44 [37] in R v3.1.1 (R Development Core Team, 2014) by running Markov Chain Monte Carlo (MCMC) methods using Just Another Gibbs Sampler (JAGS) at a time-step of eight hours. To estimate movement parameters and behavioural mode, two MCMC chains were run for 40,000 iterations with a 30,000 sample burn-in and thinned every 10 samples leaving every 10^th^ sample from the remaining 10,000 for parameter estimation. Autocorrelation was assessed visually via autocorrelation plots and chain convergence for each parameter was estimated by Gelman and Rubin’s potential scale reduction factor which was < 1.1 for all parameters signifying convergence. The BSSM calculates a continuous probability behavioural state value between 0 (transient) and 1 (resident) where a value >0.5 is presumed to exhibit resident behaviour.

Location estimates from the BSSM were then used to calculate home ranges at 50% and 95% kernel utilization distributions (KUD) using the AdehabitatHR package v0.4.13 [38] in R. Home ranges were calculated separately for each individual and then for all individuals combined. To assess spatial use relative to the proposed development, the number of locations within each 5 km distance interval from Mukkawar Island were isolated. Distance intervals from the proposed development site were established as polygons using the ‘Buffer Tool’ in ArcMap 10.2^®^ (ESRI, Redlands, California), and the number of points within each distance interval were identified using the ‘Select by Location’ function.

## Results

**Fig 2:**
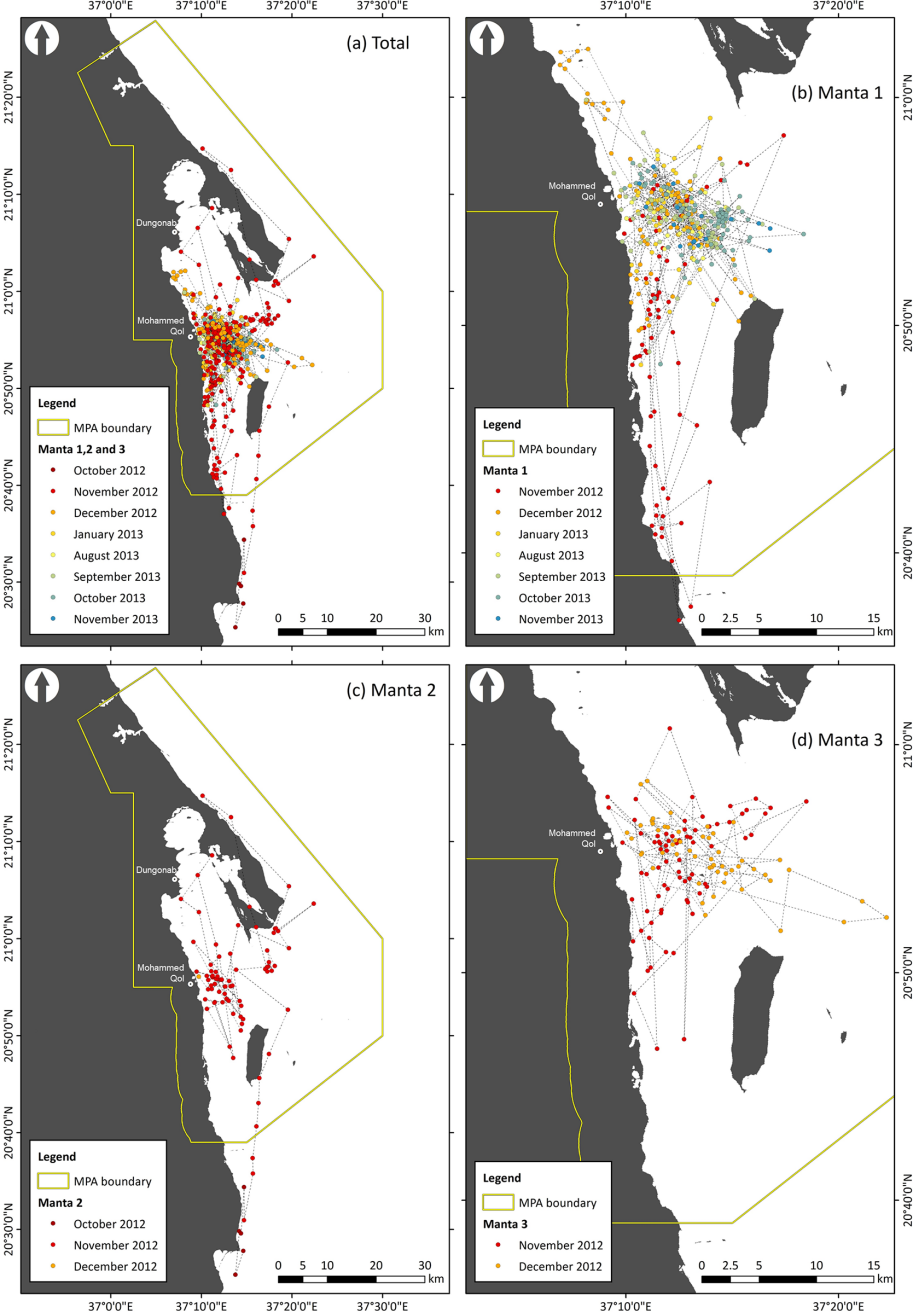
Filtered recorded locations of manta rays colour coded by month and year, for (a) total manta ray detections, (b) manta 1, (c) manta 2, and (d) manta 3, relative to the designated boundaries of the Dungonab Bay and Mukkawar Island National Park.

**Fig 3:**
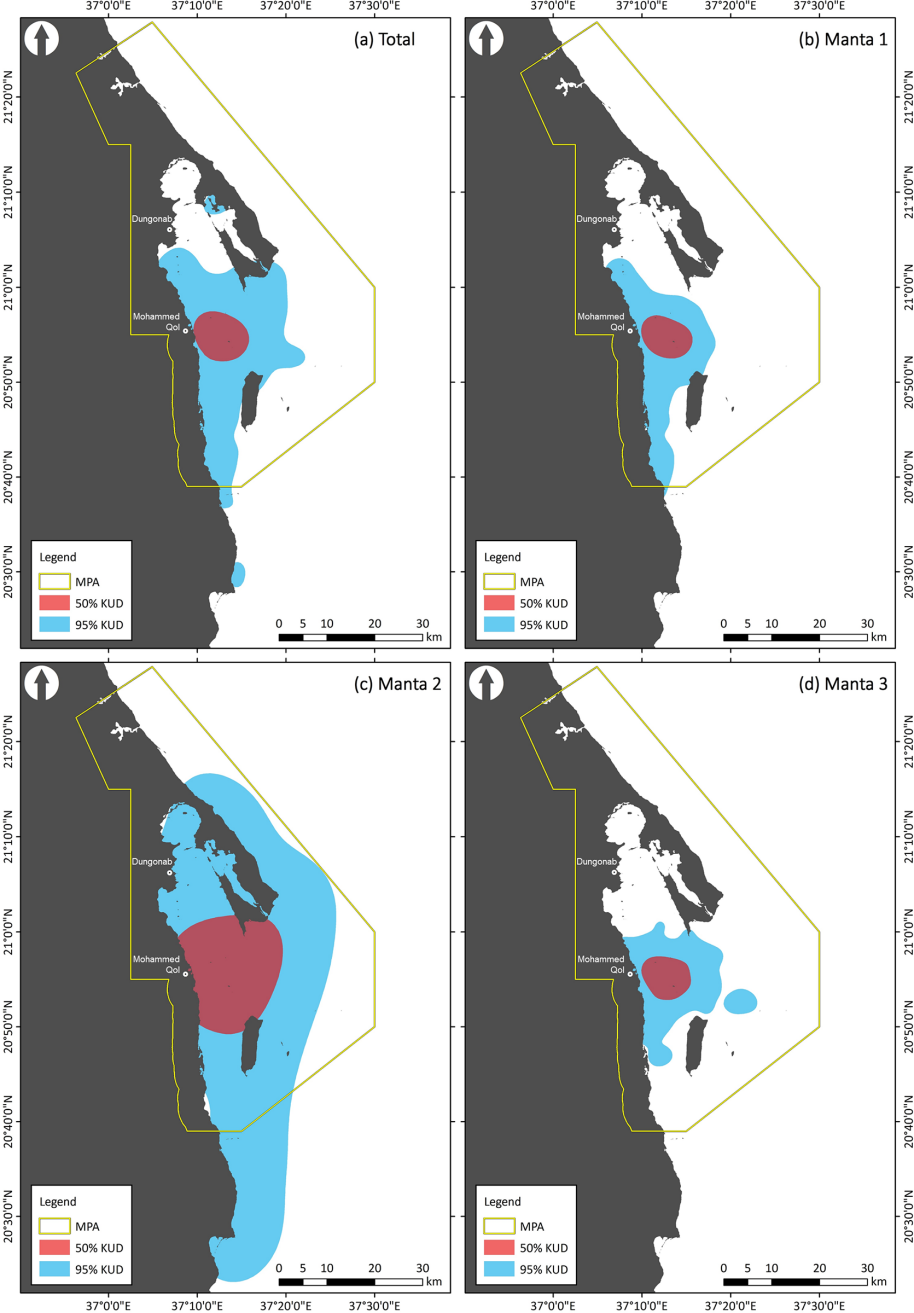
Kernel utilization distributions (KUD) home-ranges, 50% (red) and 95% (blue), for (a) total manta ray detections, (b) manta 1, (c) manta 2, and (d) manta 3, relative to the designated boundaries of the Dungonab Bay and Mukkawar Island National Park.

**Fig 4:**
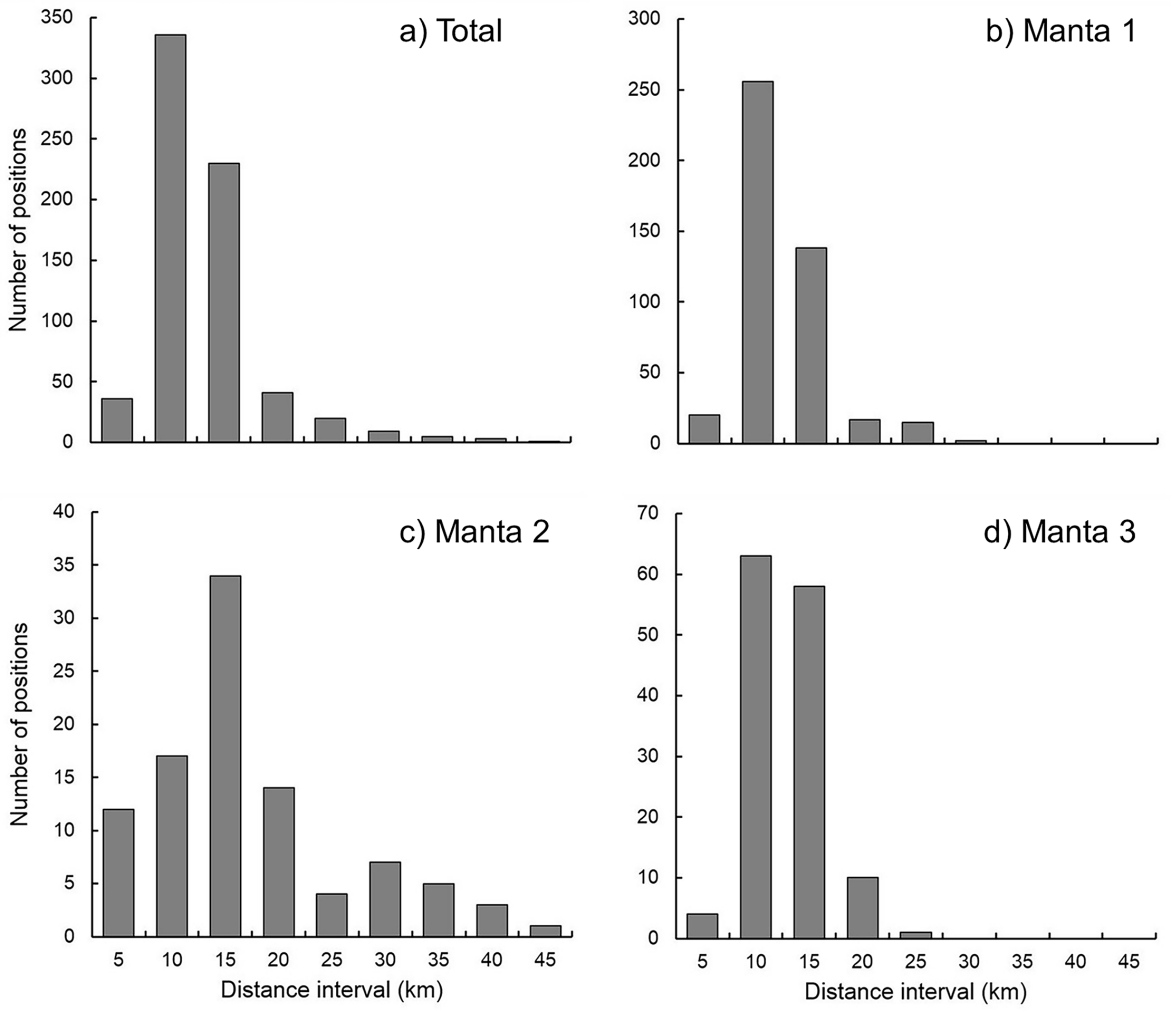
Number of detections recorded at different distance intervals from Mukkawar Island, the proposed site of a large-scale island development, (a) total manta ray detections, (b) manta 1, (c) manta 2, and (d) manta 3.

Three manta rays were fitted with SPOT 5 tags, two male and one female, ranging in size from 220 to 366 cm wingspan (Table 1). A total of 682 BSSM locations were recorded between 30 October 2012 and 6 November 2013, 448 for manta 1, 97 for manta 2, and 137 for manta 3 (Fig 2; raw unfiltered locations provided in supplementary information S5 Fig). Manta 1 was tracked over a period of 366 days with locations recorded on 149 days, manta 2 over 220 days with locations recorded on 33 days, and manta 3 over 46 days with locations recorded on all 46 days. Location were recorded from October 2012 to January 2013, and then again from August to November 2013, with no locations recorded between February and July 2013 (Fig 2; supplementary information S1 Video). Of these 682 locations, 98.5% fell within the boundaries of the MPA; 99.5% for manta 1, 91.5% for manta 2, and 100% for manta 3. The BSSM identified that all three mantas were resident/foraging for 99% of the time when transmissions were received. The 50% KUD home ranges for all three manta rays were also predominantly located within the boundaries of the MPA; the 95% KUD home ranges estimates showed a similar trend (Table 2; Fig 3). When considering the proximity of manta habitat use compared to the proposed large-scale island development, 92.4%, 64.9% and 91.9% of locations for manta rays 1, 2 and 3, respectively, occurred with 15 km of Mukkawar Island (Fig 4; 88.4% for all mantas combined).

**Table 1:** Coastal manta capture and tracking information. Locations given in decimal degrees.

ID	Date	Sex	Size (cm)	Track duration (days)	Release lat (N)	Release long (E)
Manta 1	30-Oct-12	F	366	366	20.92264	37.23957
Manta 2	27-Oct-12	M	220	32	20.85729	37.22631
Manta 3	28-Oct-12	M	300	45	20.87473	37.21672

**Table 2:** Kernel utilization distributions (KUD) home range areas for individual manta rays and all manta rays combined.

ID	50% KUD (Km^2^)	95% KUD (Km^2^)
Manta 1	63.5	491.4
Manta 2	414.1	2456.9
Manta 3	64.3	387.2
All	81.6	861.1

## Discussion

The distribution of manta ray location estimates indicated that the established boundaries of the DMNP are spatially appropriate, and identified effective MPA designation for protecting manta rays in the region. It is, however, possible that these individuals may have moved outside of the MPA while not at the surface and particularly during the period from February to July 2013 when no locations were recorded. The focused distribution of the recorded locations, including the observation that 99% of locations the mantas were deemed to be resident/foraging, suggested that the DMNP is a highly productive and important habitat for manta rays in the Red Sea region. The close proximity of the recorded locations and estimated home ranges of the tracked manta rays to Mukkawar Island highlights the potential threat of disruption to the local reef manta ray population, should the development be executed to the full extent of the proposed plans. The relative close proximity to the recorded locations to the two local Beja communities of Mohamed Qol and Dungonab demonstrates the potential to develop a small-scale ecotourism industry based on watching manta rays, that could potentially bring significant economic benefits to the people currently living inside the boundaries of the MPA.

The novel capture technique employed in this study to attach satellite tags to manta rays offered several benefits over past tagging techniques for this species. Specifically, the restraint of the animals allowed the SPOT tags to be firmly secured to the dorsal fin, resulting in extended tracking durations. Post release snorkeler observations revealed that all tagged manta rays swam off well. Additionally, tagged individuals were observed and photographed in feeding aggregation trains the following day, indicating a rapid return to normal behaviour. Although the number of individuals tagged for this study is relatively low, the longevity of the tracking and the urgent need for data from this highly understudied region highlights their importance. The manta rays tagged in this study are a subset of individuals sampled for genetics and implanted with acoustic transmitters. It is anticipated that continued investigations will further elucidate the behaviours and dynamics of this regionally important population. Future investigation will increase sample size to help validate, and further describe, the spatial ecology of manta rays in the region.

Consistent use of coastal and reef areas by reef manta rays has been documented throughout the globe, including, Mozambique [39], Hawaii [40], and Australia [12, 41]. For example, across the Red Sea, off the coast of Saudi Arabia, reef manta rays demonstrated site fidelity to specific reef sites, exhibited regional movements up to 200 km from the tagging site [42], and occupied the surface waters during the daytime and deeper waters at night [43]. At Lady Elliot Island, on the Great Barrier Reef, Australia, reef manta rays were observed to form similar feeding aggregations when zooplankton density reached a threshold of 11.2 mg m^-1^ [44]. Site fidelity of coastal mantas to a specific lagoon, similar to the data reported here, was documented through acoustic tracking at Palmyra Atoll, where observed movements and stable isotope data suggested that food availability was the primary driver for residence within the lagoon area [3]. The sheltered nature of lagoon areas can retain surface waters and increase productivity [45], providing reliable feeding opportunities and calm conditions for reef manta rays [3, 4]. Other potential benefits of lagoon areas are refuge from predators, such as large sharks [46], breeding grounds due to potential for mating opportunities and their function as nursery areas [3]. In the case of the Dungonab manta ray aggregation, the latter is a likely benefit, as juveniles and suspected neonates have been regularly sighted within the boundaries of the MPA [20].

The success of MPAs in protecting species depends largely on the spatial extent of the area protected and the spatial ecology of the target species [47]. In the case of the DMNP, reef manta rays are arguably the species with the greatest potential to make large-scale movements or migrations [3], but these data demonstrated they primarily resided inside the MPA boundaries when in the region. This is in contrast to satellite tracked pelagic manta rays around the Yucatan, Mexico, where only 11.5% of locations were recorded within MPA boundaries [48]. The effectiveness of the DMNP may be attributed to the lagoon residency, relative to the coastal distribution of the Yucatan manta rays, and the potential for pelagic manta rays to undertake large-scale pelagic movements.

Effective MPA designation has implications for both the environmental health of the ecosystem and the manta ray-associated ecotourism industry. The growing ecotourism sector of Sudan stands to substantially benefit from reliable and regular access to the manta ray aggregation. Mindful use of the manta ray aggregation for ecotourism development is important for local villages, with more ecologically sustainable tourism being championed by other government stakeholders. For example, a current initiative (21–019), supported by the Darwin Initiative and UKAid aims to develop community involvement in ecotourism surrounding the manta ray population and to stimulate employment opportunities in the region. This initiative is intended to develop appropriate scale sustainable ecotourism activities in conjunction with local communities, and, thus, minimize potential threats to traditional ways of life, as was experienced by the Bedouin communities of the Sinai Peninsula, Egypt, during the development of the scuba dive tourism industry [49]. In Egypt, the rapid influx of capital investment and western amenities associated with the tourism industry resulted in a dilution of traditional culture, which would be favorable to avoid or minimize in Sudan. In Indonesia, it is estimated that manta ray-related ecotourism generates ~US$15 million per year [11, 50]. In the Red Sea itself, it has been estimated that a single shark can be worth as much as US$200,000 in neighbouring Egypt’s scuba diving ecotourism economy, with the success of elasmobranch dive sites such as the Three Brothers [51]. Given the relative scale of operations in the Sudan, the revenue potential would be much lower than in Egypt. However, in a region of very limited employment opportunities, this natural resource represents an important economic asset.

Although these preliminary data suggest the DMNP provides legislative protection to this regional manta ray population, in reality there is a considerable lack of resources for active enforcement. This shortfall is both in terms of physical resources, namely patrol vessels, but also financial resources for operations. The WCGA of Sudan owns a vessel based in the fishing village of Dungonab and 10 wildlife office have been stationed onsite since 2016. However, due to limited resources such as fuel for the vessel, they would be largely powerless to intervene should illegal exploitation occur (WCGA pers. comms.). To date, manta ray exploitation in the region has not previously been reported, but increasing global demand for manta ray products, in particular branchial filter plates [8, 9], raises concern over future exploitation. Barring a change to marine policy and MPA designation in the country, such exploitation would be conducted illegally. The potential for exploitation is relatively high, as the country has experienced illegal fishing, mainly of Yemeni origin, targeting elasmobranchs [52]. Given these factors, the current priority for increasing the effectiveness of the MPA is increased patrols and enforcement of its protected status.

With the extent of the MPA offering spatially appropriate protection, the greatest threat to the Dungonab Bay manta ray population, aside from capture, could originate from the terrestrial environment. The proposed large-scale island development on Mukkawar Island is in close geographical proximity to the recorded reef manta ray locations, and these waters constitute an important feeding area for this manta ray population. The negative effects of coastal developments on marine ecosystems are well documented [25]. As a filter feeding species, the reef manta ray predominantly forages on lower trophic level organisms, mainly zooplankton [53]. Heavy dredging, associated with the development plans, would not only result in direct benthic habitat loss, but would also greatly increase turbidity in the surrounding waters [25]. Such turbidity drives a reduction in primary productivity and in turn zooplankton, reducing available food resources in these important feeding areas [25, 29, 30]. Turbidity resulting from dredging activities would also have negative ecosystem impacts for many organisms including corals [31, 54]. These disruptions could destabilise the local marine ecosystem and potentially displace the reef manta ray population to areas outside of the boundaries of the MPA.

During and following development completion, vessel traffic could directly disrupt the manta rays, as the most probable shipping route directly intersects the 95% KUD area for all three tracked manta rays. Secondary effects following development completion, including runoff and pollution, could also cause further ecosystem disruptions [55]. It is hoped, however, that the recent Sudanese-led designation of the DMNP as an UNESCO World Heritage Site would make it unlikely that these development plans will proceed as previously planned, but national and international awareness of this proposed development and its potential impacts is essential. With government stakeholders currently debating vastly contrasting tourism trajectories, providing important data about the spatial use of key charismatic/flagship species, such as reef manta rays, is crucial to contribute to the decision-making process and support more sustainable options.

## Supporting information

S1 Fig Satellite derived mean monthly variation in (a) sea surface temperature, (b) chlorophyll-a and (c) turbidity, as represented by K_d_(490) the diffuse attenuation coefficient at 490 nm, which is one indicator of the turbidity of the water column, for Dungonab Bay and Mukkawar Island National Park (DMNP) (from Klaus, R. 2016 Final Draft Management Plan for Dungonab and Mukkawar Island Protected Area, Sudan 2016 to 2021. Volume I Current Conditions, and Volume II Operations Manual. *World Bank GEF funded Strategic Ecosystem Management (SEM) for the Red Sea and Gulf of Aden Project*, pp. 366. PERSGA, Jeddah, Saudi Arabia).
(TIF)

S2 Fig Monthly mean chlorophyll-a concentrations (AquaMODIS) illustrating the seasonal changes in primary productivity in the Sudanese Red Sea. The white line shows the Exclusive Economic Zone (EEZ) of Sudan (source: VLIZ 2008). Values shown are the concentration of chlorophyll-a (mg/l). The data for each month is scaled separately, where blue shows low productivity and red shows high productivity for each month (from Klaus, R. 2016 Final Draft Management Plan for Dungonab and Mukkawar Island Protected Area, Sudan 2016 to 2021. Volume I Current Conditions, and Volume II Operations Manual. *World Bank GEF funded Strategic Ecosystem Management (SEM) for the Red Sea and Gulf of Aden Project*, pp. 366. PERSGA, Jeddah, Saudi Arabia).
(TIF)

S3 Fig Mean monthly turbidity, represented by K_d_(490), the diffuse attenuation coefficient at 490 nm, one indicator of water column turbidity derived from AquaMODIS satellite data. The data for each month is scaled separately such that, blue shows the lowest mean turbidity and red shows highest mean turbidity for each month. The legends show the turbidity range for each month (from Klaus, R. 2016 Final Draft Management Plan for Dungonab and Mukkawar Island Protected Area, Sudan 2016 to 2021. Volume I Current Conditions, and Volume II Operations Manual. *World Bank GEF funded Strategic Ecosystem Management (SEM) for the Red Sea and Gulf of Aden Project*, pp. 366. PERSGA, Jeddah, Saudi Arabia).
(TIF)

S4 Fig Wildlife Computers^®^ SPOT 5 tag being attached to the dorsal fin of a reef manta ray (*Manta alfredi*) using nylon bolts. The reef manta ray is secured to the side of the vessel with ropes.
(TIF)

S5 Fig Raw location data received from reef manta rays, colour coded by location accuracy from excellent (red– 3) to poor (blue–B). (TIF)

S1 Video Supplementary video file “Kessel et al filtered locations over time.avi” depicts the distribution of filtered location records by day across the study period (October 2012 to November 2013). Locations are colour coded by individual–manta 1 (green), manta 2 (pink), and manta 3 (yellow).
(AVI)

S1 Data Supplementary data file “Kessel et al raw manta SPOT tag data.csv” contains raw unfiltered SPOT 5 tag data received from all three manta rays. Manta 1 (PTT 122197), manta 2 (PTT 122198), and manta 3 (PTT 122199).
(CSV)
