# Supplementary figures and images for: Conservation of reef manta rays (Manta alfredi) in a UNESCO World Heritage Site: Large-scale island development or sustainable tourism?
Source: PLoS One. 2017 Oct 25;12(10):e0185419. doi: 10.1371/journal.pone.0185419 (PMC5656316; doi:10.1371/journal.pone.0185419)

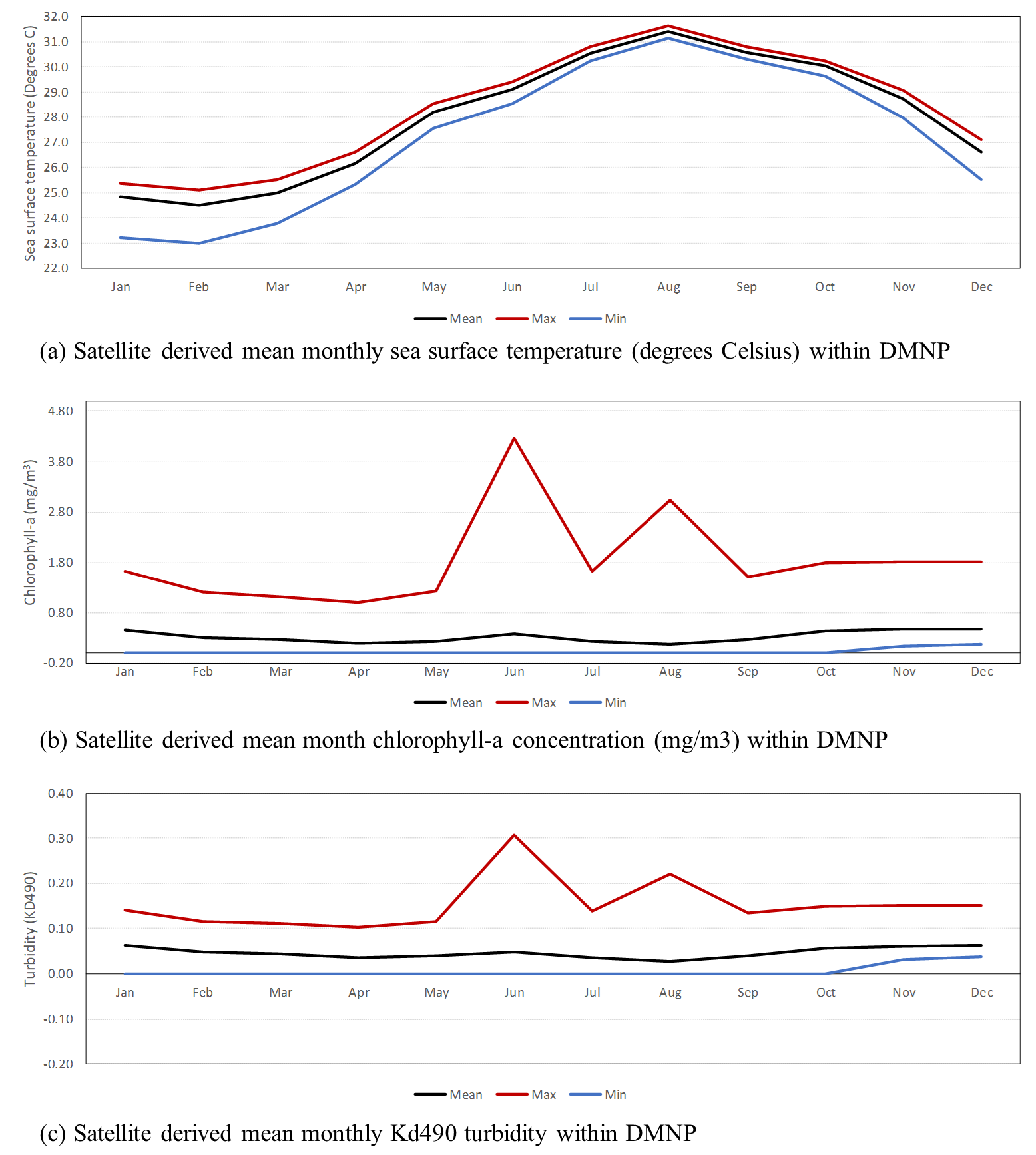

Supplement: S1 Fig — Satellite derived mean monthly variation in (a) sea surface temperature, (b) chlorophyll-a and (c) turbidity, as represented by Kd(490) the diffuse attenuation coefficient at 490 nm, which is one indicator of the turbidity of the water column, for Dungonab Bay and Mukkawar Island National Park (DMNP) (from Klaus, R. 2016 Final Draft Management Plan for Dungonab and Mukkawar Island Protected Area, Sudan 2016 to 2021. Volume I Current Conditions, and Volume II Operations Manual. World Bank GEF funded Strategic Ecosystem Management (SEM) for the Red Sea and Gulf of Aden Project, pp. 366. PERSGA, Jeddah, Saudi Arabia). (TIF) [file pone.0185419.s001.tif]

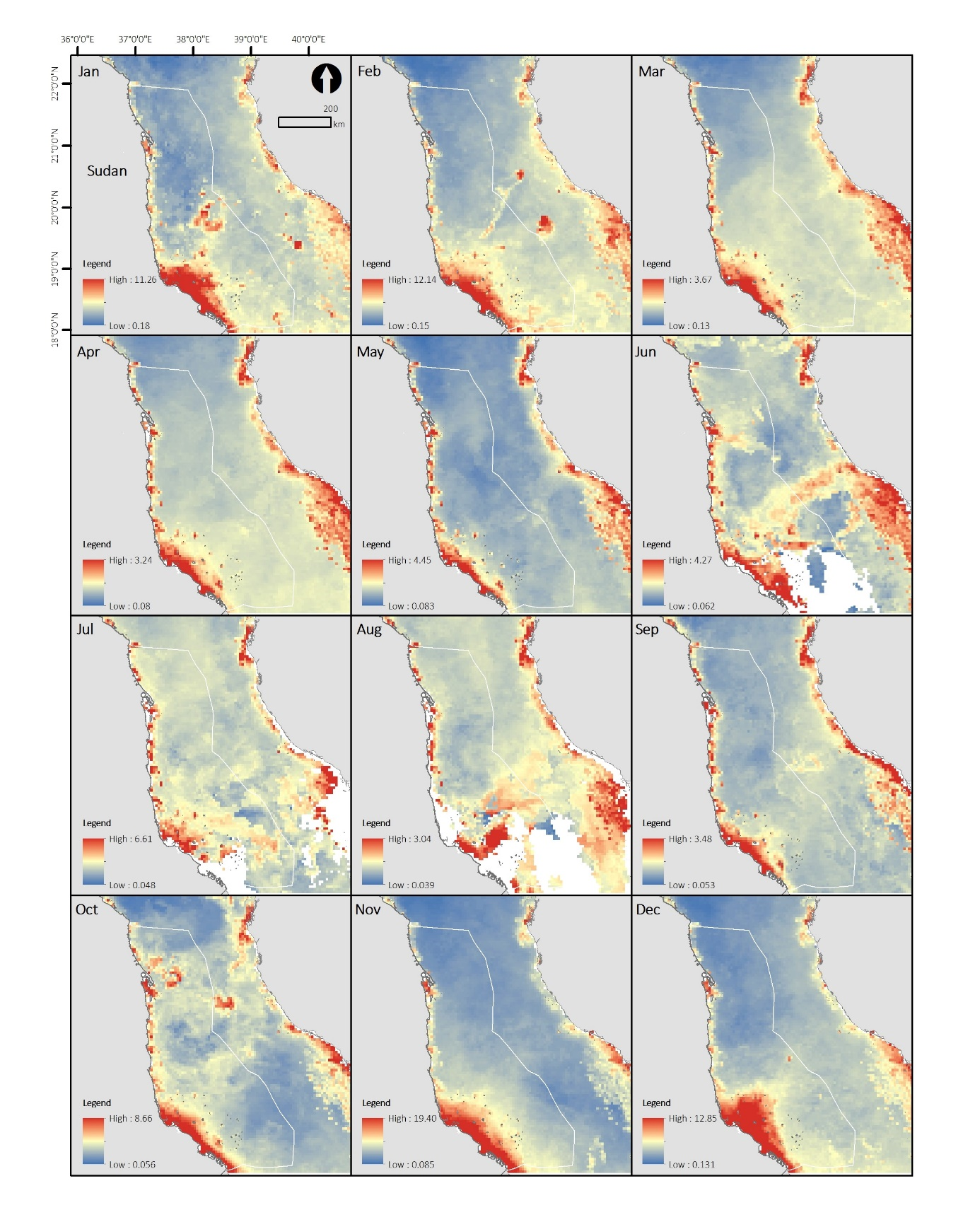

Supplement: S2 Fig — The white line shows the Exclusive Economic Zone (EEZ) of Sudan (source: VLIZ 2008). Values shown are the concentration of chlorophyll-a (mg/l). The data for each month is scaled separately, where blue shows low productivity and red shows high productivity for each month (from Klaus, R. 2016 Final Draft Management Plan for Dungonab and Mukkawar Island Protected Area, Sudan 2016 to 2021. Volume I Current Conditions, and Volume II Operations Manual. World Bank GEF funded Strategic Ecosystem Management (SEM) for the Red Sea and Gulf of Aden Project, pp. 366. PERSGA, Jeddah, Saudi Arabia). (TIF) [file pone.0185419.s002.tif]

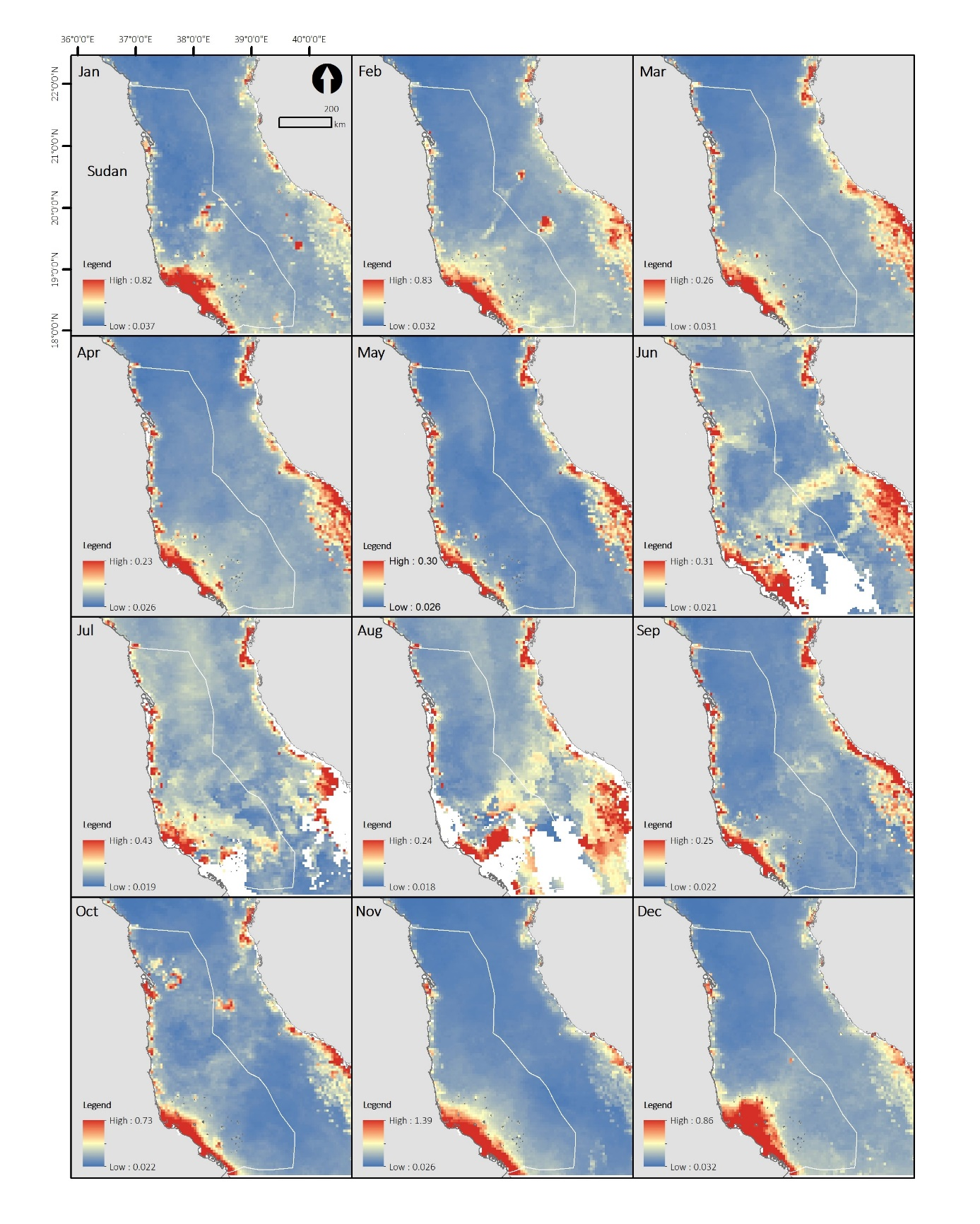

Supplement: S3 Fig — The data for each month is scaled separately such that, blue shows the lowest mean turbidity and red shows highest mean turbidity for each month. The legends show the turbidity range for each month (from Klaus, R. 2016 Final Draft Management Plan for Dungonab and Mukkawar Island Protected Area, Sudan 2016 to 2021. Volume I Current Conditions, and Volume II Operations Manual. World Bank GEF funded Strategic Ecosystem Management (SEM) for the Red Sea and Gulf of Aden Project, pp. 366. PERSGA, Jeddah, Saudi Arabia). (TIF) [file pone.0185419.s003.tif]

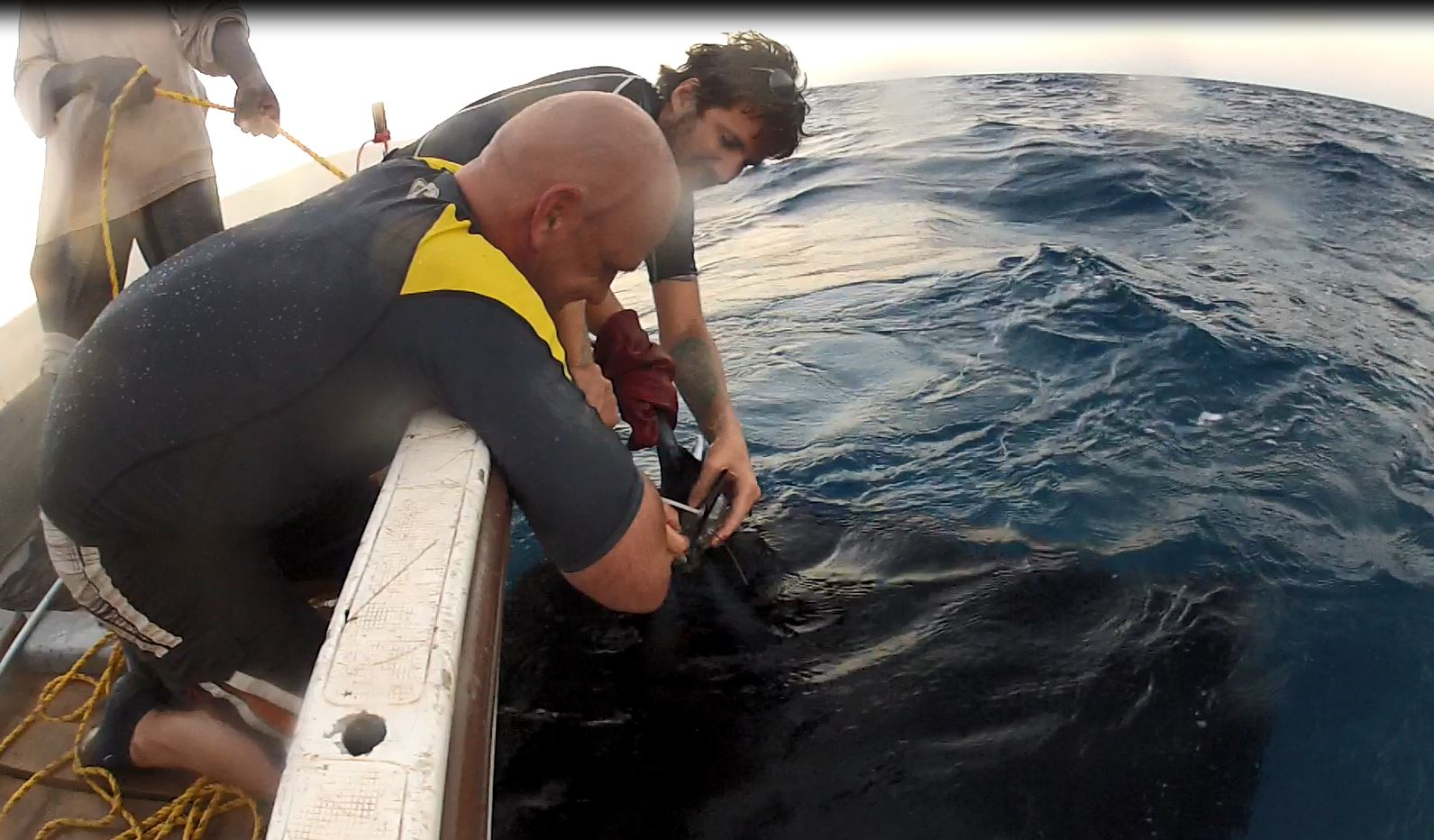

Supplement: S4 Fig — The reef manta ray is secured to the side of the vessel with ropes. (TIF) [file pone.0185419.s004.tif]

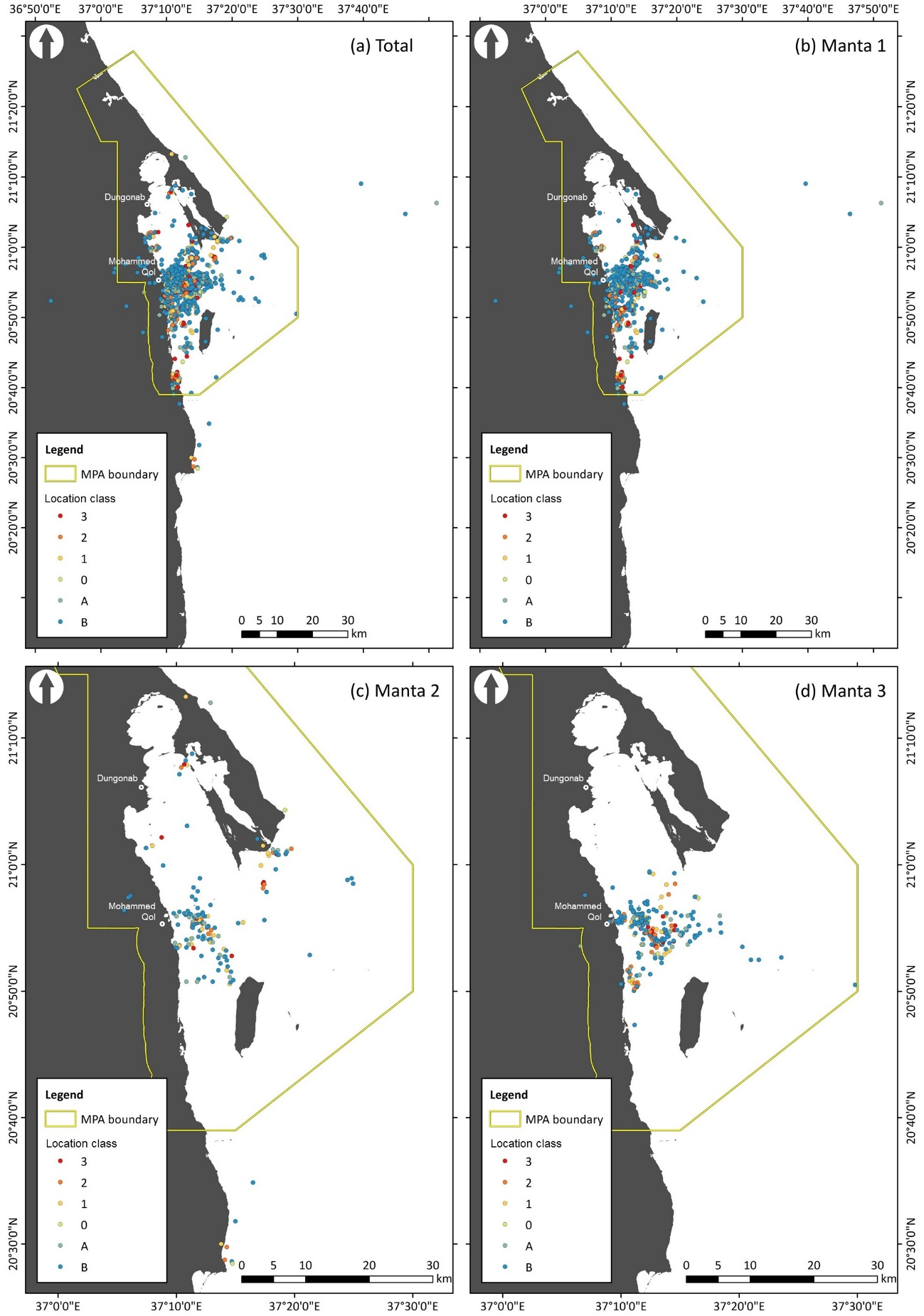

Supplement: S5 Fig — (TIF) [file pone.0185419.s005.tif]
